# Single-cell transcriptomics and machine learning unveil ferroptosis features in tumor-associated macrophages: Prognostic model and therapeutic strategies for lung adenocarcinoma

**DOI:** 10.3389/fphar.2025.1598756

**Published:** 2025-05-12

**Authors:** Ting Ji, Juanli Jiang, Xin Wang, Kai Yang, Shaojin Wang, Bin Pan

**Affiliations:** ^1^ Department of Key Laboratory of Ningxia Stem Cell and Regenerative Medicine, Institute of Medical Sciences, General Hospital of Ningxia Medical University, Yinchuan, Ningxia, China; ^2^ Department of Pulmonary and Critical Care Medicine, General Hospital of Ningxia Medical University, Yinchuan, Ningxia, China; ^3^ Department of Neurology, The First Affiliated Hospital of Chengdu Medical College, Chengdu, Sichuan, China; ^4^ School of Clinical Medicine, Chengdu Medical College, Chengdu, Sichuan, China; ^5^ Department of Respiratory and Critical Care Medicine, The First Affiliated Hospital of Chengdu Medical College, Chengdu, Sichuan, China

**Keywords:** ferroptosis, lung adenocarcinoma (LUAD), macrophage, prognosis, ferroptosis of macrophage, single-cell transcriptome analysis

## Abstract

**Background:**

Lung adenocarcinoma (LUAD) is a major cause of cancer-related mortality worldwide. Tumor-associated macrophages (TAMs) play a crucial role in the tumor microenvironment (TME), influencing tumor progression and immune response. Ferroptosis, an iron-dependent form of regulated cell death, has been implicated in tumor biology, but its role within TAMs in LUAD remains unclear.

**Aim:**

This study aimed to screen key genes associated with ferroptosis in macrophages and construct a prognostic risk model for LUAD based on these genes.

**Methods:**

Integrating the TCGA-LUAD, GSE131907, and GSE13213 datasets, macrophage heterogeneity was analyzed through single-cell dimensionality reduction clustering, pseudotime analysis, and cell-cell communication. Using the GeneCards ferroptosis gene set (1515 genes), ferroptosis-related differentially expressed genes in macrophages were screened. Eight machine learning algorithms (LASSO, SVM, XGBoost, etc.) were leveraged to identify prognostic genes and build a Cox regression risk model. The functional roles of key genes were validated through immune infiltration analysis, drug sensitivity prediction, and Western blot analysis.

**Results:**

Single-cell analysis revealed that macrophages in LUAD lead intercellular communication through the MIF (CD74+CXCR4) ligand-receptor interaction, with ferroptosis-related genes (FRGs) highly expressed in macrophages. 73 macrophage FRGs were identified, and through multi-algorithm cross-validation, HLF, HPCAL1, and NUPR1 were determined as core genes. The risk model (Risk Score = HLF × (−0.153) + HPCAL1 × 0.261 + NUPR1 × (−0.21)) demonstrated robust predictive performance in both the TCGA and GSE13213 cohorts, with 1-, 3-, and 5-year AUC values of 0.756, 0.753, and 0.705. The high-risk group was enriched in tumor progression pathways (like epithelial-mesenchymal transition, cell cycle checkpoints), exhibited low expression of immune checkpoint genes (BTLA, CD47), and showed increased sensitivity to cyclophosphamide and crizotinib. Western blotting confirmed the expression levels of HLF, HPCAL1, and NUPR1 were remarkably lower in LUAD cell lines compared to normal bronchial epithelial cells (P < 0.05).

**Conclusion:**

The research is the first to build a LUAD prognostic model based on macrophage ferroptosis-related genes (HLF, HPCAL1, NUPR1), revealing the immune microenvironment characteristics and drug sensitivity differences in the high-risk group. These findings provide new strategies for precision therapy targeting ferroptosis in tumor-associated macrophages (TAMs).

## 1 Introduction

Lung cancer (LC) is among the most prevalent malignancies globally and a leading cause of cancer-related mortality (Sung et al., 2021; Oncology Branch of Chinese Medical AssociationChinese Medical Association Publishing House, 2023). Lung adenocarcinoma (LUAD), the predominant histological subtype, accounts for approximately 40% of all lung cancer cases (Siegel et al., 2024). The management of LUAD necessitates individualized treatment strategies tailored to disease progression, with surgery being the primary modality for early-stage disease, while systemic therapies are employed for advanced stages (Hirsch et al., 2017; Kris et al., 2017). Despite treatment advancements, overall survival rates for LUAD remain suboptimal, particularly the concerning 5-year survival figures (Yang et al., 2023; Jeon et al., 2023). There is growing evidence to suggest that therapeutic strategies solely targeting LUAD tumor cells possess limited efficacy in inhibiting disease progression or enhancing treatment outcomes.

The tumor microenvironment (TME), including non-tumor cells such as TAMs, is a current research hotspot and plays a significant role in tumor development. TAMs promote tumor cell proliferation, migration, and metastasis by secreting cytokines and growth factors, while also altering the tumor immune microenvironment and suppressing immune surveillance, which ultimately affects the response to cancer therapies (Pan et al., 2020; DeNardo and Ruffell, 2019; Liang et al., 2022; Mantovani et al., 2017). Additionally, macrophages significantly impact tumor cell drug resistance by regulating iron metabolism and promoting ferroptosis pathways (Dong et al., 2019). Therefore, targeting TAMs within the TME may represent a promising strategy for future LUAD treatment.

TAMs are the most abundant stromal cell population in the TME, mainly consisting of the classically activated M1 phenotype (anti-tumor) and the selectively activated M2 phenotype (pro-tumor) (Anderson and Simon, 2020; Zheng et al., 2020; Wu et al., 2020). M1 macrophages induce inflammatory responses by secreting pro-inflammatory cytokines such as IL-6, IL-1β, and TNF-α, which not only affect the activity of enzymes involved in iron, lipid, and amino acid metabolism (Han et al., 2021; Shanmugam et al., 2015), but also activate related metabolic pathways through generating reactive oxygen species (ROS), thereby altering the state of tissue cells and macrophages themselves (Yuan et al., 2019; Zhang J. et al., 2019; Kapralov et al., 2020; Yang et al., 2022). Ferroptosis, a new form of programmed non-apoptotic cell death, features the accumulation of iron-dependent lipid peroxides, causing cell membrane rupture and cell death (Dixon et al., 2012). Recent studies have shown that oxidative stress-induced release of KRAS^G12D^ protein by cancer cells induces autophagy-dependent ferroptosis in the TME, a process that drives macrophage polarization toward the M2 phenotype, thus promoting pancreatic cancer growth (Dai et al., 2020). On the other hand, iron overload enhances the expression of M1 macrophage markers (like IL-6, TNF-α, IL-1β) and inhibits the levels of M2 macrophage markers (such as TGM2), promoting the polarization of M1 macrophages (Handa et al., 2019). Therefore, the interaction between ferroptosis and macrophages not only regulates the balance of the tumor immune microenvironment but may also influence immune evasion mechanisms in tumors by affecting iron metabolism and oxidative stress responses. However, the specific impact of ferroptosis in macrophages on the TME, especially in terms of tumor infiltration and patient prognosis, remains incompletely understood. Further investigation into the regulatory role of ferroptosis in macrophages within the TME will not only provide new insights into the immune microenvironment of tumors such as LUAD but may also open new therapeutic strategies for precision oncology.

However, despite increasing research interest in macrophages and ferroptosis, substantial knowledge gaps persist regarding the specific roles and molecular mechanisms of macrophage ferroptosis in the progression of tumors, particularly LUAD, and in the remodeling of the tumor microenvironment (TME). For instance, how macrophage ferroptosis precisely modulates the functions of downstream immune cells and tumor cell behavior remains unclear. Furthermore, the key regulatory genes and associated signaling pathways governing macrophage ferroptosis are yet to be fully elucidated. These unresolved questions impede the potential development of targeting macrophage ferroptosis as a novel therapeutic strategy for LUAD. Consequently, an in-depth investigation into the regulatory network of ferroptosis within macrophages and its functional implications in the LUAD microenvironment is crucial for advancing our understanding of LUAD immune regulatory mechanisms and identifying new therapeutic targets.

Single-cell sequencing technologies provide unprecedented resolution for dissecting the complexity and cellular heterogeneity of the TME. This study innovatively integrates multi-center LUAD single-cell transcriptome datasets (n = 3) to systematically analyze the cellular heterogeneity of TME. Through single-cell dimensionality reduction clustering, we constructed a spatial distribution map of immune and stromal cells within the TME, focusing on the functional characteristics of macrophage subpopulations. Pseudotime trajectory analysis revealed the dynamic differentiation pathways of macrophages, and ligand-receptor interaction networks were used to decipher their cellular communication patterns, leading to the identification of key genes regulating the ferroptosis process. Based on these critical genes, a prognostic risk score model was built, systematically assessing the relationship between different risk groups, patient prognosis, and the immune microenvironment. This model not only effectively predicts patient survival but also provides potential therapeutic strategies for targeting the tumor immune microenvironment. Finally, through drug sensitivity analysis, we further validated the model’s applicability in chemotherapy drug selection, offering scientific evidence for precision treatment. By combining single-cell transcriptomics with multi-omics analysis, this study provides new insights and strategies for immune microenvironment regulation and personalized precision therapy in LUAD.

## 2 Materials and methods

### 2.1 Acquisition and preprocessing of bulk RNA-seq data

In this study, we utilized The Cancer Genome Atlas (TCGA) database and the Bioconductor package ‘TCGAbiolinks’ (v2.25.0) to download whole-genome expression data and related clinical information for LUAD, with the expression data provided in TPM (Transcripts Per Million) format. The TCGA-LUAD dataset (n = 600) includes 541 tumor samples and 59 normal control samples (https://www.cancer.gov/ccg/research/genome-sequencing/tcga). Additionally, we obtained the GSE13213 dataset from the GEO database, encompassing 117 LUAD samples. During data preprocessing (https://www.ncbi.nlm.nih.gov/geo/), samples with missing survival time or survival time of zero were excluded to ensure all analyzed samples had valid prognostic information. Furthermore, samples with more than 50% missing gene expression data were excluded, and genes not expressed in more than 50% of the samples were also removed.

### 2.2 Download and processing of single-cell RNA sequencing data

We obtained the single-cell RNA sequencing dataset GSE131907 from the GEO database (https://www.ncbi.nlm.nih.gov/geo/). This dataset encompasses 11 tumor tissue samples, 11 distant normal lung tissue samples, 10 normal lymph node samples, and 10 metastatic brain tissue samples, all from untreated patients who underwent conservative surgery. Additionally, the dataset contains seven metastatic lymph node samples, four lung tumor samples, and five samples from malignant pleural effusion of advanced LUAD patients. The data were processed via the ‘Seurat’ package (version 5.1.0) and normalized using the ‘normalizeData’ function. The initial quality control (QC) included the calculation of complexity scores (log10GenesPerUMI = log10 (nFeature_RNA)/log10 (nCount_RNA)) and mitochondrial gene ratios (mitoRatio), computed using the PercentageFeatureSet function with the pattern '^MT-'. Cells were filtered based on the following criteria: 500 < nCount_RNA <5,000, nFeature_RNA >200, log10GenesPerUMI >0.9, and mitoRatio <0.2. Genes expressed in fewer than 100 cells were excluded. In addition, we performed comprehensive quality control procedures: potential doublets were identified and removed using the scDblFinder package; batch effects across samples were corrected using the Harmony algorithm to minimize technical variation; and cell cycle effects were regressed out during data normalization and scaling to reduce confounding influences.

High-variance genes were selected based on the mean expression and dispersion, and 19 cell clusters were generated on 30 principal components (PCs) using the ‘FindClusters’ algorithm, optimized by the shared nearest neighbor (SNN) module, with a resolution set to 0.8. Subsequently, t-SNE analysis was performed using the ‘RunTSNE’ algorithm to visualize cell clustering. Differentially expressed genes (DEGs) were identified via the ‘FindAllMarkers’ function in Seurat, and cell clusters were annotated while assessing the proportions of different cell types.

### 2.3 AUC score analysis of ferroptosis-related genes (FRGs)

We retrieved 1,515 FRGs from the GeneCards database (https://www.genecards.org/). Gene set enrichment analysis (GSEA) was carried out via the ‘AUCell’ package, and the AUC values for each cell were calculated to assess the expression levels of FRGs. Cells with higher AUC values indicate higher expression levels of FRGs. The AUC scores were visualized using a UMAP plot, displaying the spatial distribution of cells within different score groups.

### 2.4 Cell communication analysis

We utilized the ‘CellChat’ package (version 1.6.1) to analyze intercellular communication, with a focus on the interactions of macrophages with other cell types. CellChat calculates ligand-receptor pairs and their associated signaling pathways to reveal the intercellular communication network. The results were visualized via heatmaps and bubble plots, displaying the enrichment of ligand-receptor pairs and the strength of communication between different cell types. Additionally, CellChat was used to analyze the relative strength distribution of endogenous and exogenous signaling pathways across different cell types, revealing how different cell types respond to external signals in the tumor microenvironment, such as immune and inflammatory responses.

### 2.5 Pseudotime analysis

We performed pseudotime analysis using the ‘Monocle 2’ tool on genes with high expression variance and high expression levels (variance ≥1, average expression ≥0.1), constructing a pseudotime trajectory plot for the cells. This analysis revealed the developmental trajectories of LUAD cells and illustrated the changes in cell states. Branch expression analysis modeling (BEAM) was used to further analyze gene expression changes during cell fate decisions. The gene expression differences across various trajectory branches were visualized using heatmaps. Specifically, an independent pseudotime ordering trajectory analysis was conducted on macrophages, revealing their development and differentiation within the LUAD microenvironment.

### 2.6 Identification of DEGs

We used the ‘limma’ package (Liu S. et al., 2021) to identify DEGs between early-stage LUAD and normal samples. The Benjamini–Hochberg method was applied for multiple testing correction, and the adjusted p-values (adj.p-value) were calculated. DEGs with FDR <0.05 and |log2FC| ≥ 1 were selected. Subsequently, the ‘clusterProfiler’ package (Yu et al., 2012) was leveraged to perform enrichment analysis of these DEGs, False discovery rate (FDR) correction was performed using the Benjamini–Hochberg method, with a selection criterion of FDR <0.05.

### 2.7 Identification of FRGs in macrophages

Through Venn diagram analysis, we identified the intersecting genes between macrophage-related genes and FRGs in LUAD. Subsequently, we analyzed the differential expression of these intersecting genes between tumor and normal tissues via the TCGA-LUAD dataset, ultimately identifying the differentially expressed FRGs in macrophages in LUAD.

### 2.8 Construction and validation of the prognostic risk model

To comprehensively and robustly identify ferroptosis-related macrophage genes significantly associated with prognosis, we implemented an integrated strategy combining eight machine learning algorithms based on distinct principles. These included regularization-based methods (e.g., LASSO), well-suited for feature selection in high-dimensional data; kernel-based approaches (e.g., SVM), capable of capturing nonlinear relationships; tree-based ensemble methods (e.g., Random Forest, XGBoost, and Bagging), known for their robustness and ability to model complex interactions; and other techniques such as Boruta, which focuses on identifying all relevant features, and Learning Vector Quantization (LVQ). To mitigate algorithm-specific bias and enhance the reliability and reproducibility of the selected genes, we intersected the outputs from these algorithms and retained genes identified by at least three of them. This multi-algorithm ensemble approach is widely adopted in biomarker discovery and recognized for its improved predictive power and generalizability (Shao X, et al. Transl Psychiatry, 2024) (Shao et al., 2024).

Following the identification of candidate genes through this multi-algorithm strategy, statistical methods were employed to construct a prognostic model. First, univariate Cox regression analysis was performed for each candidate gene to preliminarily assess its association with overall survival. Genes demonstrating a significant association (P < 0.05) were then included in a multivariate Cox regression analysis. Stepwise selection was applied to determine the final set of prognostic genes and their corresponding regression coefficients. Variance Inflation Factor (VIF) analysis was also conducted to assess multicollinearity among the selected genes. These Cox regression analyses were conducted using the ‘survminer’ R package.

A prognostic risk model was then constructed based on the selected genes and their coefficients derived from the multivariate Cox regression analysis in the TCGA training cohort. The RiskScore for each sample was calculated using the formula: RiskScore = β1X1 + β2X2 + . + βnXn (where β represents the regression coefficients, and X represents the gene expression values). Subsequently, samples from both the TCGA training cohort and the GEO validation cohort were stratified into high-risk (HRG) and low-risk groups (LRG) based on the median of their calculated RiskScores. The prognostic performance of the model was evaluated using Kaplan-Meier survival analysis, comparing the survival rates between the high- and low-risk groups, and the predictive accuracy of the RiskScore was assessed by plotting Receiver Operating Characteristic (ROC) curves.

### 2.9 Correlation analysis between clinical features and RiskScore

We integrated clinical data from early-stage LUAD patients and analyzed the distribution differences between various clinical features (such as age, TNM stage, gender, smoking history, etc.) and RiskScore.

### 2.10 Construction of the nomogram

By combining clinical feature data and RiskScore, univariate and multivariate Cox regression analyses were performed to identify independent prognostic factors. A Nomogram model was then constructed using these factors. The rms package was utilized for analysis and plotting to establish a Nomogram for predicting the 1-year, 3-year, and 5-year overall survival of early-stage LUAD patients. The predictive ability of the model was validated through calibration curves.

### 2.11 TME analysis

We leveraged the ‘CIBERSORT’ and ‘ssGSEA’ algorithms to analyze the differences in immune cell infiltration between the HRG and LRG. Additionally, the ‘ESTIMATE’ package was used to compute the immune score, stromal score, and ESTIMATE score for each group, further exploring the relationship of RiskScore with the immune microenvironment. This analysis helps to reveal the potential impact of RiskScore on the tumor immune environment, particularly in terms of immune cell infiltration and tumor immune evasion.

### 2.12 GSEA and GOKEGG analysis

We applied GSEA to carry out enrichment analysis of hallmark gene sets (c2.all.v2024.1.Hs.symbols) as well as Hub gene-associated GOKEGG pathways between RiskScore groups. The selection criteria were p-value <0.05 and |NES| > 1 to ensure the significance of the enrichment results. Through these analyses, we further explored the functional and signaling pathway differences between RiskScore groups, revealing pathways closely associated with tumorigenesis, immune responses, and cellular metabolism processes.

### 2.13 Immune treatment response analysis

We evaluated the differences in tumor mutational burden (TMB) among patients in different RiskScore groups and calculated the cytokine gene expression (CYT) scores. Additionally, we analyzed the expression differences of immune checkpoint genes. Through these metrics, we explored the relationship of RiskScore with immune treatment response, with a particular focus on the potential role of immune evasion mechanisms in the HRG. False discovery rate (FDR) correction was performed using the Benjamini–Hochberg method, with a selection criterion of FDR <0.05. This analysis provides valuable insights into the potential effectiveness of immune therapy.

### 2.14 Drug sensitivity analysis

Using the GDSC database, we assessed the sensitivity of patients in different RiskScore groups to common targeted therapies and chemotherapeutic agents. The ‘oncoPredict’ package was used to quantify the IC50 values for each drug, and drugs with significant differences between HRG and LRG were selected. This analysis provides valuable insights for personalized treatment in LUAD patients, particularly in the clinical application of selecting targeted therapies and chemotherapeutic agents.

### 2.15 Cell culture

BEAS-2B cells (C6106, Beyotime, CN) were cultured in RPMI-1640 medium (10–040-CVR, Corning, CN) supplemented with 10% fetal bovine serum (FBS-F-500, CytoBiotech, FR) and 1% penicillin-streptomycin (PS-100X, CytoBiotech, FR) at 37°C in a 5% CO_2_ atmosphere. For subculturing, cells were rinsed with pre-cooled PBS and then digested with 0.25% trypsin-EDTA for 2 min. NCI-H1975 (CL-0298, Procell, CN) and A549 (CL-0016, Procell, CN) cells were cultured in RPMI-1640 medium containing 10% FBS. Digestion times were 3–5 min for NCI-H1975 cells and 1–2 min for A549 cells.

### 2.16 Western blot (WB) analysis

Total protein was extracted using RIPA lysis buffer containing protease and phosphatase inhibitors (50 mM Tris-HCl, pH 7.4, 1% Triton X-100). Protein concentration was determined by the BCA method (A65453, Thermo Fisher, US), with 30 μg loaded per lane.

Proteins were separated by 12% SDS-PAGE and transferred onto PVDF membranes (ISEQ00010, Millipore, US). Membranes were blocked with 5% non-fat milk at room temperature for 1 h.

Primary Antibody Incubation: The following primary antibodies were diluted 1:1000 and incubated overnight at 4°C: HLF (ab317427, Abcam, United Kingdom), NUPR1 (ab6028, Abcam, United Kingdom), HPCAL1 (IPD-ANP11286, ABclonal, CN) Secondary Antibody Incubation: Membranes were incubated with horseradish peroxidase (HRP)-conjugated secondary antibodies at room temperature for 1 h. Signal Detection: Detection was performed using ECL, and results were recorded with the Bio-Rad Chemidoc XRS + imaging system.

## 3 Results

### 3.1 Single-cell transcriptomic analysis

#### 3.1.1 Single-cell dimensionality reduction, clustering, and annotation

**FIGURE 1:**
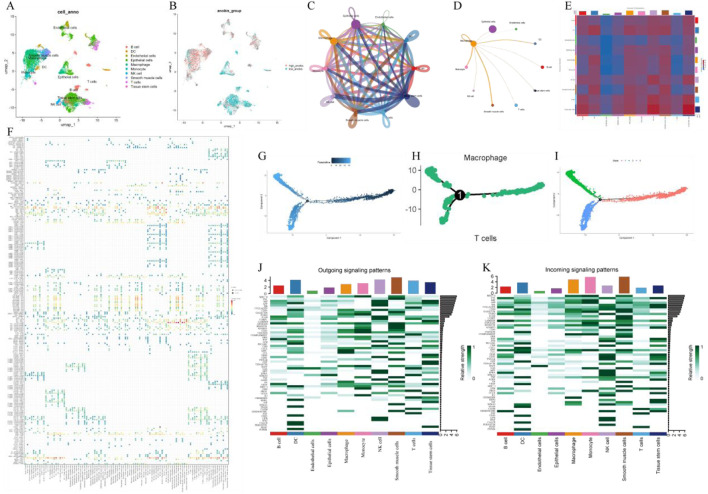
Single-cell transcriptome analysis. **(A)** UMAP plot of cell clustering; **(B)** UMAP plot of AUC scores for ferroptosis-related genes; **(C)** Single-cell communication interaction diagram; **(D)** Macrophage communication interaction diagram. **(E)** Heatmap of intercellular communication; **(F)** Bubble plot of intercellular communication; **(G)** Pseudotime trajectory analysis; **(H)** Hierarchical pseudotime trajectory analysis; **(I)** Pseudotime trajectory of macrophages; **(J)** Relative intensity distribution of exogenous signaling pathways across different cell types; **(K)** Relative intensity distribution of endogenous signaling pathways across different cell types.

We analyzed gene expression profiles of LUAD cell populations using the GSE131907 dataset, comprising 11 tumor and 11 distant normal lung tissue samples. Through t-SNE analysis, these cells were divided into 19 clusters, which were subsequently annotated based on cell-specific markers and expression patterns. A total of 10 cell types were identified, including epithelial cells, macrophages, as well as T cells (Figure 1A). Additionally, AUC score analysis based on ferroptosis-related genes revealed higher expression of these genes in smooth muscle cells and macrophages (Figure 1B).

#### 3.1.2 Pseudo-time analysis

In the identified macrophages, we constructed a pseudo-time cell trajectory to explore key gene expression programs in LUAD progression. Through pseudo-time analysis, we successfully built the pseudo-time trajectory of macrophages, revealing distinct transcriptional states and cell fate decision processes (Figures 1G–I).

#### 3.1.3 Cell communication analysis

To further elucidate the integrated roles of various cell types in lung cancer tissues, we performed intercellular communication analysis (Figures 1C, E) as well as macrophage-centric communication analysis (Figure 1D). The results suggested macrophages, as the primary signal providers, exhibited the strongest interactions with other cell types via the MIF (CD74 + CXCR4) ligand-receptor pair (Figure 1F). Macrophages play a bidirectional role in both outward and inward signaling, participating in immune responses through outward signals and engaging in intercellular interactions via strong responses to external signals. Notably, macrophages displayed strong responses in both outward and inward signaling in pathways such as EGF and CCL (Figures 1J, K). These preliminary results provide compelling support for a deeper understanding of the integrated functions of macrophages in LUAD.

### 3.2 Identification of ferroptosis-related genes in macrophages associated with LUAD

**FIGURE 2:**
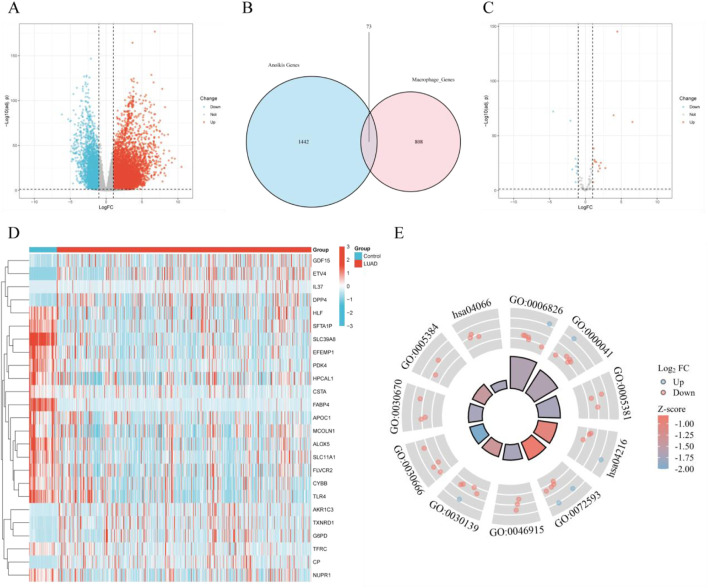
Selection of differentially expressed genes (DEGs) and identification of ferroptosis-related genes. **(A)** Volcano plot of DEGs in the TCGA database; **(B)** Venn diagram of macrophage-related genes and ferroptosis-related genes; **(C)** Volcano plot of differentially expressed genes at the intersection of macrophage-related and ferroptosis-related genes in the TCGA database; **(D)** Heatmap of differentially expressed genes at the intersection of macrophage-related and ferroptosis-related genes; **(E)** Combined GOKEGG and fold change (FC) circos plot of intersecting differentially expressed genes in macrophages and ferroptosis.

By screening for DEGs between normal lung tissue and LUAD tissue, we identified 14,953 DEGs, of which 11,657 genes were upregulated and 3,296 genes were downregulated (Figure 2A). Further single-cell differential analysis of LUAD tissue revealed 881 macrophage-related genes. Additionally, we retrieved 1,515 ferroptosis-related genes from the GeneCards database. By taking the intersection of these two sets, we identified 73 ferroptosis-related genes within macrophages (Figure 2B). A subsequent differential analysis of these genes led to the selection of 25 filtered genes (Figure 2C). The expression patterns of these genes are shown in the heatmap (Figure 2D). GO and KEGG enrichment analyses of the filtered genes suggested these genes may impact several biological processes in cells, including iron ion transport (GO:0006826), fatty acid metabolism (GO:0030670), apoptosis (GO:0072593), and cancer-related signaling pathways (hsa04066), among others. These enrichment results provide important insights for further investigation into cellular responses under various physiological or pathological conditions (Figure 2E).

### 3.3 Machine learning-based selection of key ferroptosis-related genes in macrophages of LUAD

**FIGURE 3:**
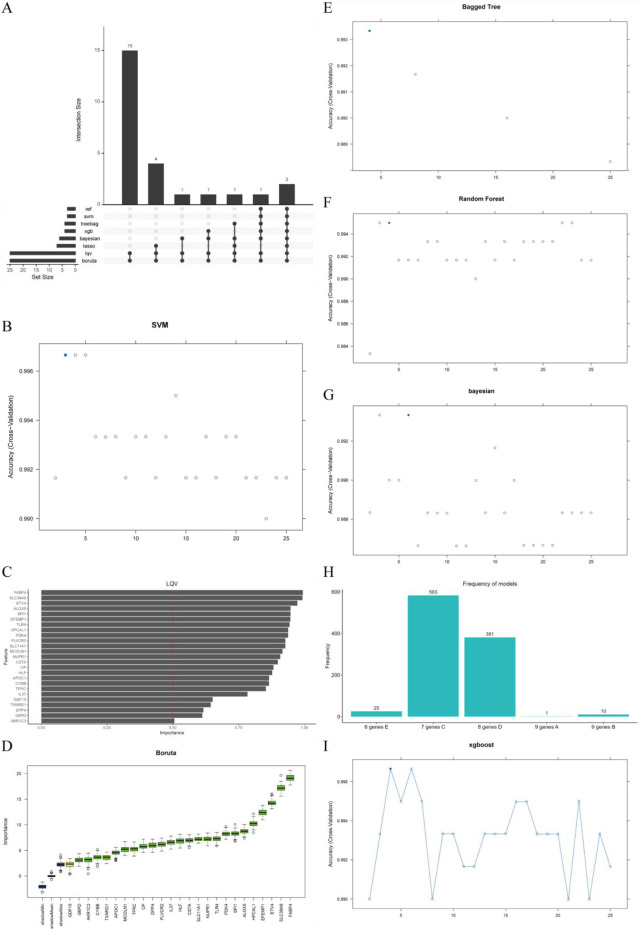
Selection of prognosis-related genes and identification of diagnostic genes. **(A)** Intersection size of eight machine learning algorithms; **(B)** Accuracy of the support vector machine (SVM) model; **(C)** LQV feature importance assessment; **(D)** Boruta feature selection results; **(E)** Accuracy of the bagged tree model; **(F)** Accuracy of the random forest model; **(G)** Accuracy of the Bayesian model; **(H)** Frequency distribution of LASSO model feature selection; **(I)** Accuracy of the XGBoost model.

We employed eight machine learning algorithms (RFE, LASSO, RF, SVM, GBDT, Bagging, XGBoost, and Boruta) to further screen the 25 filtered genes (Figure 3A). Using the SVM algorithm, we identified three optimal feature genes (Figure 3B). The LQV algorithm validated all included genes with a cutoff of 0.5 (Figure 3C). The Boruta algorithm also validated all included genes and displayed the changes in z-scores (Figure 3D). The Bagging decision tree selected four genes (Figure 3E). RF, combined with feature selection, eliminated 22 genes based on the number of classification trees and error rates (Figure 3F). The Bayesian algorithm selected six predictive genes (Figure 3G). The LASSO logistic regression method selected seven predictive genes from statistically significant univariate analyses (Figure 3H). The XGBoost algorithm identified four genes (Figure 3I). Ultimately, we selected 10 key genes, which were recognized by at least three models, for further analysis.

### 3.4 Construction and validation of the prognostic risk model

**FIGURE 4:**
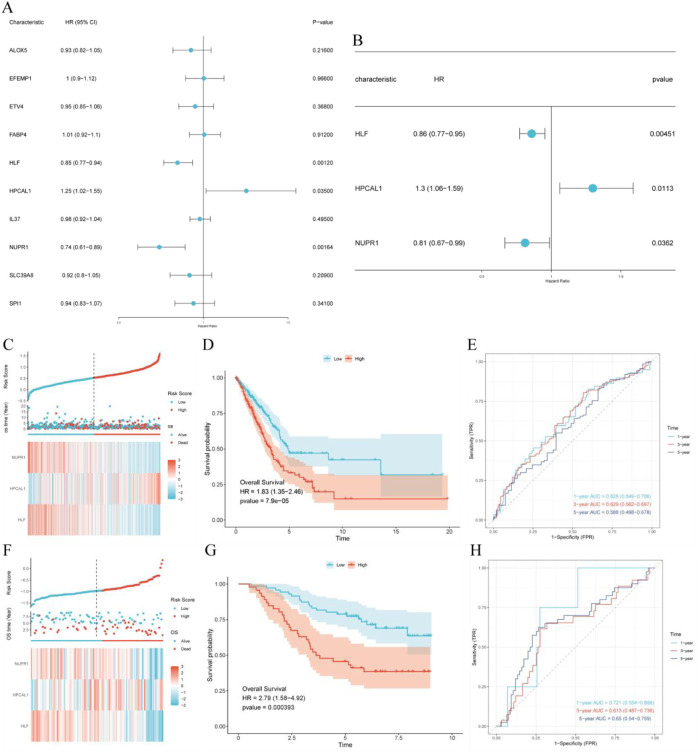
Construction and validation of the prognostic risk model. **(A)** Univariate Cox regression analysis of intersecting genes; **(B)** Multivariate Cox regression analysis of intersecting genes; **(C)** TCGA cohort: Risk score, survival distribution, and heatmap of gene expression; **(D)** Kaplan-Meier survival curve of the TCGA cohort; **(E)** Time-dependent ROC curve of the TCGA cohort; **(F)** GSE13213 cohort: Risk score, survival distribution, and heatmap of gene expression; **(G)** Kaplan-Meier survival curve of the GSE13213 cohort; **(H)** Time-dependent ROC curve of the GSE13213 cohort.

Univariate and multivariate Cox regression analyses were carried out on the key genes selected by machine learning, identifying three hub genes associated with prognosis (Figures 4A, B). Using these hub genes, we constructed a risk score (RiskScore) formula: RiskScore = HLF * (−0.153) + HPCAL1 * 0.261 + NUPR1 * (−0.21). The survival status, risk scores, as well as time-dependent ROC curves of high and low-risk score groups in both the TCGA training cohort and the GSE13213 validation cohort exhibited robust predictive performance (Figures 4C–H).

### 3.5 Expression of hub genes

**FIGURE 5:**
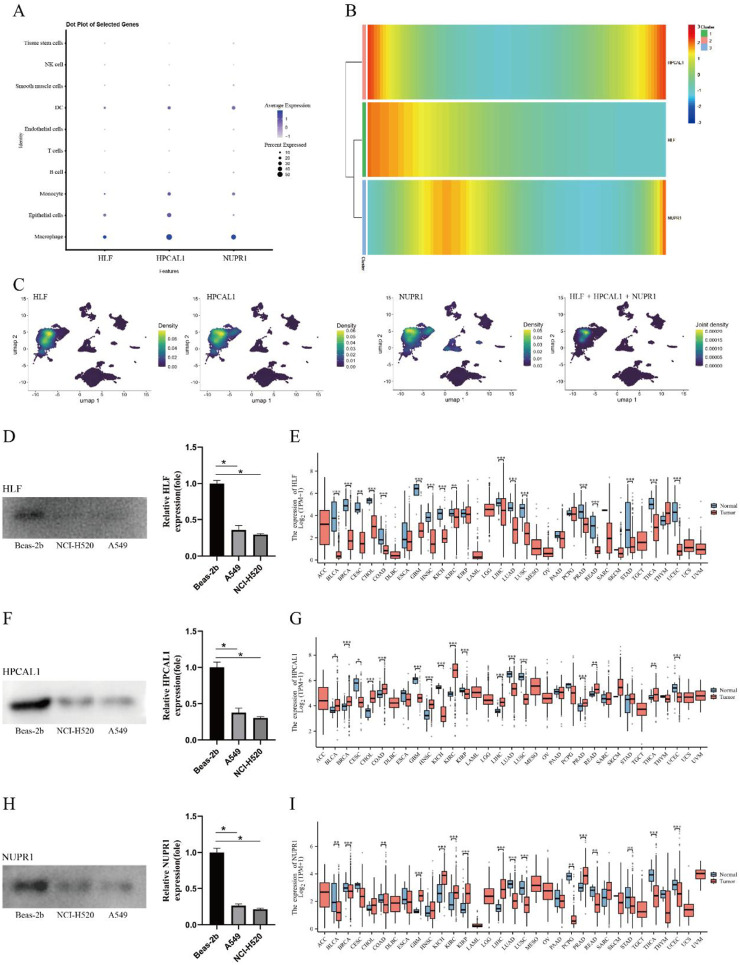
Expression patterns of hub genes. **(A)** Dot plot of hub genes; **(B)** Pseudotime heatmap of hub genes; **(C)** UMAP plot of hub gene expression; **(D)** Differential expression of HLF in NCI-H1975, A549, and BEAS-2B cell lines; **(E)** Expression levels of HLF in different cancer types and normal tissues; **(F)** Differential expression of HPCAL1 in NCI-H1975, A549, and BEAS-2B cell lines; **(G)** Expression levels of HPCAL1 in different cancer types and normal tissues; **(H)** Differential expression of NUPR1 in NCI-H1975, A549, and BEAS-2B cell lines; **(I)** Expression levels of NUPR1 in different cancer types and normal tissues.

The expression of hub genes was displayed in dot plots and UMAP plots, revealing that HLF, HPCAL1, and NUPR1 are highly expressed in macrophages and epithelial cells, suggesting that they may play important roles in immune-related functions. The expression levels in other cell types were lower, indicating the potential significance of these genes in specific cell types (Figures 5A, C). Through pseudo-time heatmap analysis, we revealed the expression trends of HLF, HPCAL1, and NUPR1 in the cell populations, and the dynamic characteristics of these genes in response to time or conditions. The heatmap clustering results highlighted the similarities and differences in gene expression across different cell populations, suggesting that these genes may play key roles in specific biological processes (Figure 5B). Analysis of data from 33 common cancers in the TCGA database revealed that the genes HLF, HPCAL1, and NUPR1 exhibit significant differential expression between tumor and normal tissues in LUAD and several other cancers. Notably, these expression differences vary across cancer types (Figures 5E, G, I).

Western blot analysis confirmed significantly lower expression levels of HLF, NUPR1, and HPCAL1 in NCI-H1975 and A549 cancer cells (p < 0.05) (Figures 5D, F, H), suggesting these genes may be involved in the development of LUAD.

### 3.6 Correlation between clinical features and RiskScore

**FIGURE 6:**
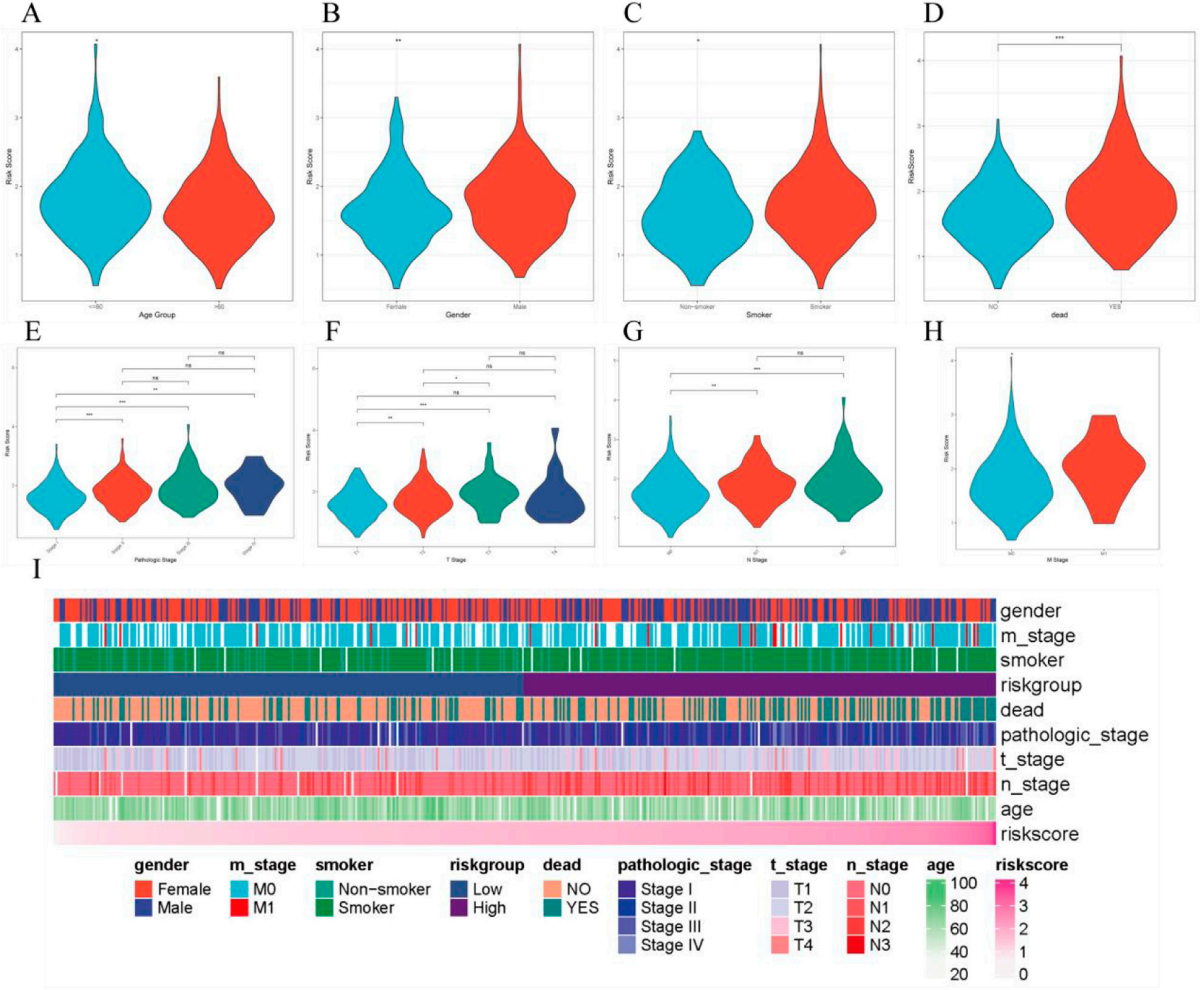
Correlation between RiskScore and various clinical features. **(A–H)**. Distribution of RiskScore across different clinical characteristics; **(I)**. Heatmap of clinical characteristics and RiskScore.

By combining clinical data from LUAD patients, we analyzed the distribution differences of RiskScore across different clinical factors. The results showed significant differences in RiskScore with respect to age, gender, smoking history, mortality, and TNM staging (Figures 6A–H). Additionally, we constructed a heatmap of RiskScore in relation to clinical factors to further explore its clinical significance (Figure 6I).

### 3.7 Construction of the nomogram

**FIGURE 7:**
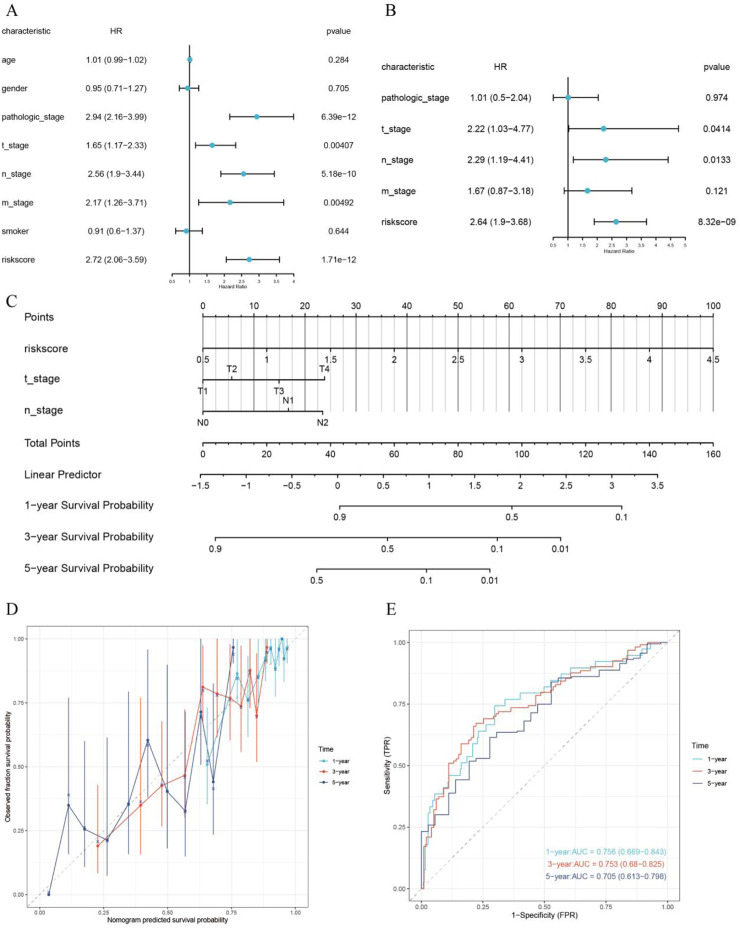
Construction and validation of the prognostic nomogram. **(A)** Univariate Cox regression analysis; **(B)** Multivariate Cox regression analysis; **(C)** Prognostic nomogram; **(D)** Calibration curve; **(E)** Time-dependent ROC curve for evaluating the diagnostic performance of the nomogram.

Through univariate and multivariate Cox regression analyses, RiskScore, T stage, as well as N stage were identified as independent prognostic factors (p < 0.05) (Figures 7A, B). A nomogram was then generated, combining RiskScore, T stage, N stage, as well as the total score, to predict the 1-year, 3-year, and 5-year survival probabilities of LUAD patients (Figure 7C). The lower the total score of independent prognostic factors, the greater the likelihood of survival for the patient. Calibration curves demonstrated that the nomogram had high accuracy in predicting survival probability in LUAD patients (Figure 7D). The time-dependent ROC curves of the nomogram model integrating T stage, N stage, and RiskScore demonstrated good predictive performance, with AUC values of 0.756, 0.753, and 0.705 for 1-year, 3-year, and 5-year survival predictions, respectively (Figure 7E).

### 3.8 TME analysis

**FIGURE 8:**
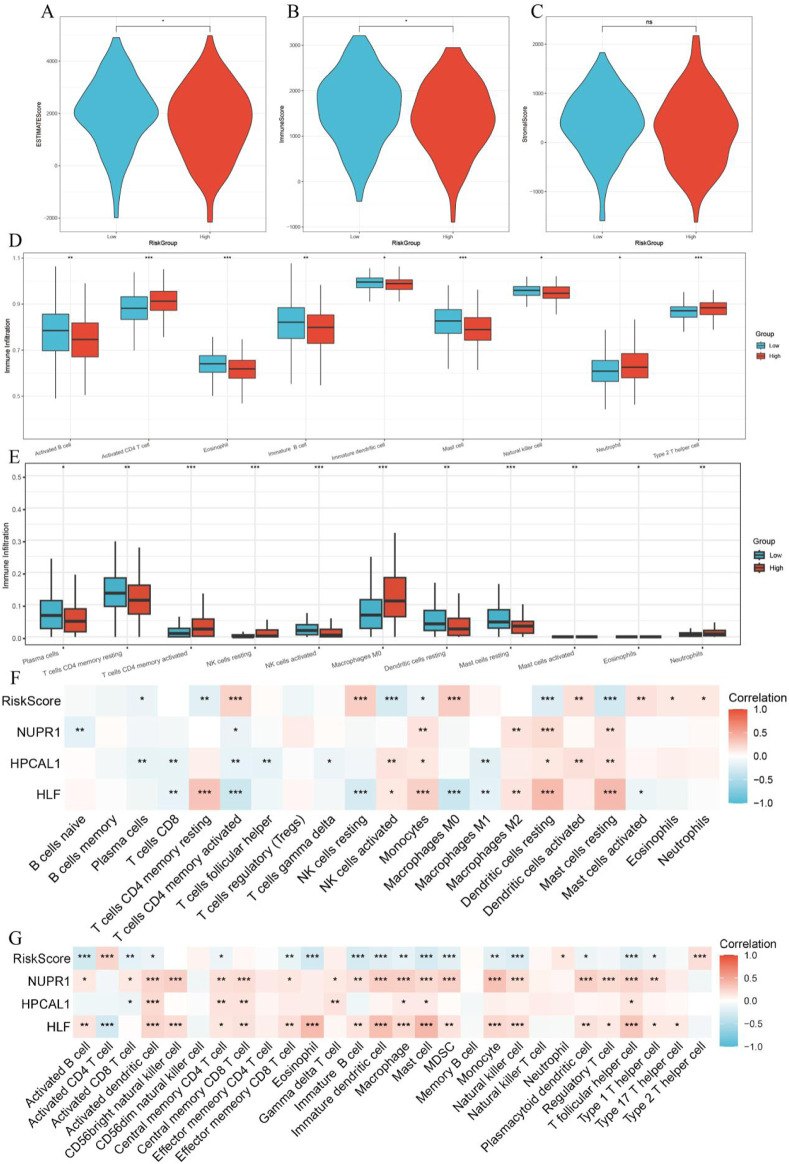
Relationship between tumor microenvironment (TME) and RiskScore. **(A–C)** Differences in StromalScore, ImmuneScore, and ESTIMATEScore between high-risk and low-risk groups; **(D)** Immune cell infiltration analysis based on the ssGSEA algorithm; **(E)** Immune cell infiltration analysis based on the CIBERSORT algorithm; **(F)** Correlation between RiskScore, three key genes, and immune cells in the CIBERSORT algorithm; **(G)** Correlation between RiskScore, three key genes, and immune cells in the ssGSEA algorithm.

The immune score calculated by the ESTIMATE algorithm was higher in the LRG, while the ESTIMATE score was higher in the HRG (p < 0.05, Figures 8A–C). Using the ‘ssGSEA’ algorithm, nine different immune cell types were identified between the two risk groups, including Activated B cells, Activated CD4 T cells, Eosinophils, Mast cells, Natural Killer cells, and others (Figure 8D). Immune infiltration analysis using the CIBERSORT algorithm showed that in the low-risk group, Plasma cells, CD4 memory resting T cells, Activated NK cells, and Resting Mast cells were more prevalent, whereas the high-risk group exhibited higher abundance of Activated CD4 memory T cells, M0 Macrophages, and Neutrophils (p < 0.05, Figure 8F).

### 3.9 GSEA analysis, GOKEGG analysis, and immune checkpoint analysis

**FIGURE 9:**
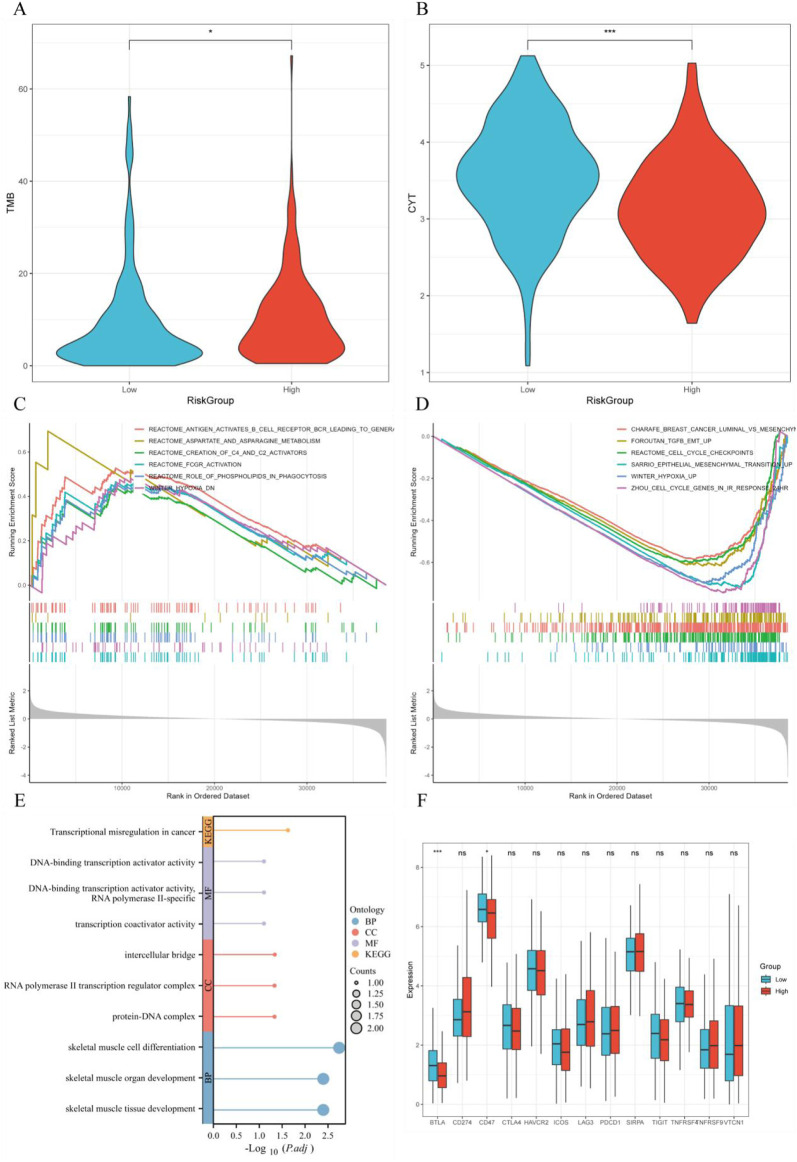
GSEA analysis, GOKEGG analysis, and immune checkpoint analysis. **(A)** Tumor mutation burden (TMB) distribution across different RiskScore groups; **(B)** Cytokine gene expression (CYT) distribution across different RiskScore groups; **(C, D)** GSEA analysis comparing high-risk and low-risk groups; **(E)** GOKEGG analysis of hub genes; **(F)** Differential expression of immune checkpoint genes across different RiskScore groups.

The high-risk group (HRG) exhibited higher TMB and lower cytotoxicity (CYT), which may be associated with tumor malignancy and immune evasion mechanisms (Figures 9A, B). GSEA analysis revealed significant enrichment in multiple signaling pathways in the HRG, particularly in pathways related to tumor progression, like immune response pathways including REACTOME_FCGR_ACTIVATION (FCγ receptor activation), REACTOME_ANTIGEN_ACTIVATES_B_CELL_RECEPTOR_BCR_LEADING_TO_GENERATION_OF_SECOND_MESSENGERS (Antigen activation of B cell receptor BCR leading to second messenger generation), cell cycle pathways like REACTOME_CELL_CYCLE_CHECKPOINTS (cell cycle checkpoints), ZHOU_CELL_CYCLE_GENES_IN_IR_RESPONSE_24HR (cell cycle genes in radiation response), and tumor cell proliferation pathways such as SARRIO_EPITHELIAL_MESENCHYMAL_TRANSITION_UP (epithelial-mesenchymal transition upregulation), REACTOME_ROLE_OF_PHOSPHOLIPIDS_IN_PHAGOCYTOSIS (role of phospholipids in phagocytosis) (Figures 9C, D). The samples with higher RiskScore may be closely associated with these more active tumor progression pathways. Additionally, the GOKEGG analysis of hub genes revealed their enrichment in multiple biological pathways, including Transcriptional Misregulation in Cancer, DNA-binding Transcription Activator Activity, and Transcription Coactivator Activity, all of which regulate gene expression in cancer cells (Figure 9E). Immune checkpoint gene expression analysis found that immune checkpoint genes, such as BTLA and CD47, were remarkably downregulated in the HRG (p < 0.05), suggesting the HRG may respond better to immune therapy (Figure 9F).

### 3.10 Chemotherapy and targeted drug IC50 analysis

**FIGURE 10:**
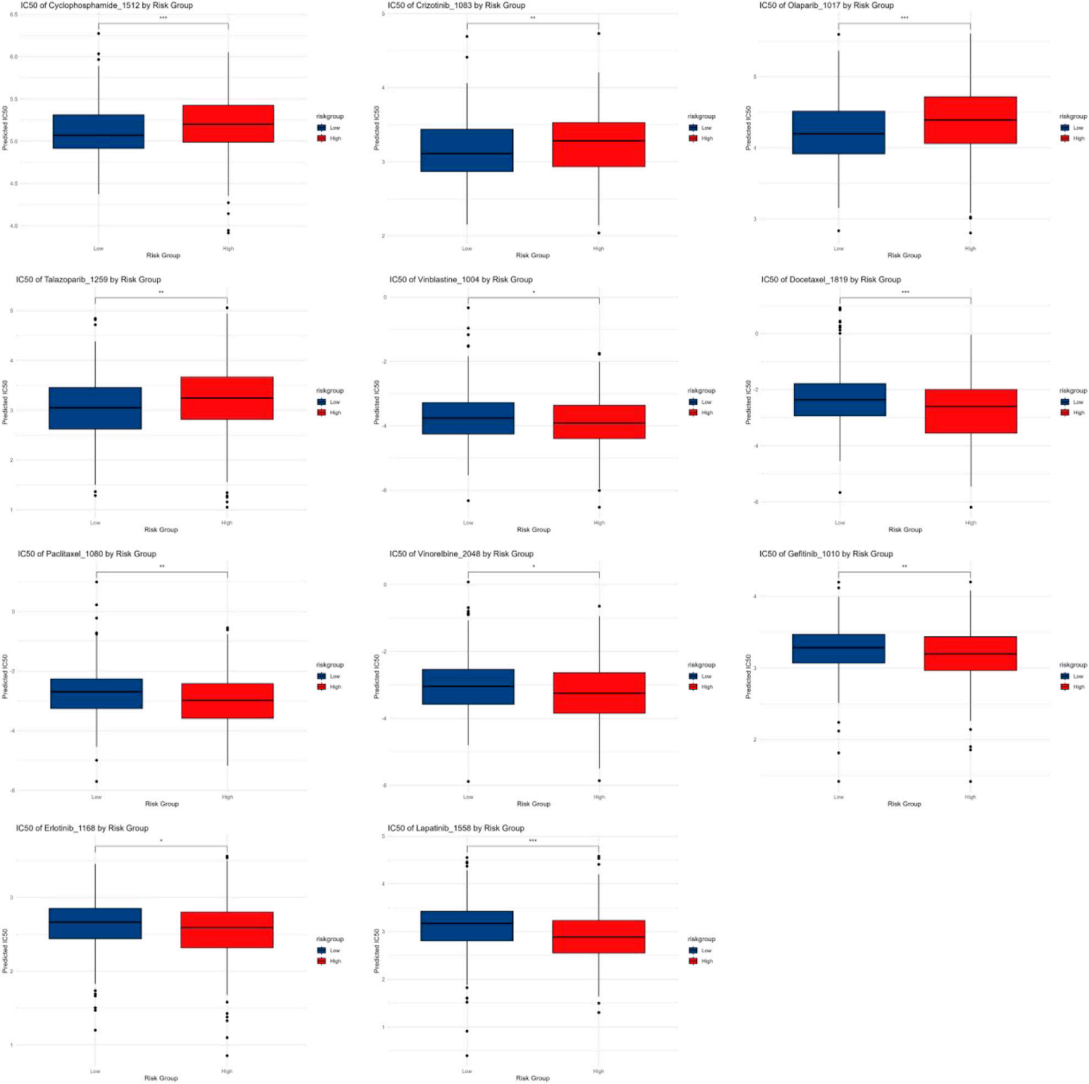
Chemotherapy and targeted drug IC50 analysis.

The IC50 analysis results demonstrated variations in drug sensitivity between patients in different RiskScore groups for common chemotherapy and targeted drugs. The results suggested the HRG exhibited higher sensitivity to chemotherapy drugs like Cyclophosphamide and targeted drugs like Crizotinib, whereas the low-risk group showed greater sensitivity to chemotherapy drugs including Vinorelbine, Paclitaxel, Docetaxel, Vinblastine, and targeted drugs like Erlotinib and Gefitinib (Figure 10).

## 4 Discussion

This study first analyzed the gene expression characteristics of LUAD cell populations and ultimately identified 10 cell types, encompassing epithelial cells, macrophages, and T cells. Through trajectory analysis and intercellular communication analysis, the developmental trajectory of macrophages was revealed, and it was found that they exhibit strong responses to both outward and inward signaling in pathways such as EGF and CCL. Research by Wang Y. et al. (2023) has shown that macrophages are the most abundant cell type in LUAD, and their impact on cancer progression varies greatly, depending on their phenotype in TME (Mantovani et al., 2002). M1 macrophages are primarily involved in pro-inflammatory responses, whereas M2 macrophages mainly participate in anti-inflammatory responses (Yunna et al., 2020). By downregulating the EGFR signaling pathway, the cannabinoid receptor two agonist JWH-015 prevents M2 macrophage-induced epithelial-to-mesenchymal transition (EMT) in NSCLC cells (Ravi et al., 2016). Additionally, M2 macrophages can upregulate anti-inflammatory cytokines and chemokines, encompassing IL-10, TGF-β, and CCL family chemokines (e.g., CCL17, CCL18, CCL22, and CCL24) (Biswas et al., 2013). In this study, AUC score analysis further revealed FRGs were highly expressed in smooth muscle cells and macrophages, suggesting a significant role for ferroptosis in these cell populations.

Ferroptosis is a form of cell death triggered by iron-dependent lipid peroxidation (LPO). Research by Hao et al. (2022) found inhibiting APOC1 promotes the conversion of M2 macrophages to M1 macrophages through the ferroptosis pathway, reshaping the tumor immune microenvironment and enhancing the response of hepatocellular carcinoma (HCC) to PD1 immunotherapy. The plasticity of macrophages highlights macrophage reprogramming as an attractive therapeutic strategy, enabling these cells to adjust their functions to meet the demands of tumor defense. In recent years, the connection between macrophages, ferroptosis, and cancer has garnered increasing attention. However, prior studies have mostly concentrated on the interaction between macrophages and ferroptosis within tumor tissues, with few addressing the impact of ferroptosis in macrophages on TME. Hence, this research presents a new perspective, aims to highlight the significance of ferroptosis-related gene signatures within TAMs on TME status and patient prognosis, which may offer complementary insights for understanding LUAD therapeutic strategies and predicting outcomes.

This study identified 14,953 DEGs between the normal and early-stage LUAD groups and extracted 1,515 ferroptosis-related genes from the GeneCards database. Through intersection analysis and differential expression analysis, 25 filtered genes were ultimately selected. GO and KEGG enrichment analyses of these genes indicated their potential roles in various biological processes, encompassing iron ion transport, fatty acid metabolism, apoptosis, and the regulation of cancer-related signaling pathways. Since LUAD is often diagnosed at an advanced stage, it is essential to identify potential biomarkers for early diagnosis and prognosis. In recent years, multiple prognostic gene signatures for lung cancer have been identified. For instance, a linear prognostic model comprising eight genes (DLGAP5, KIF11, RAD51AP1, CCNB1, AURKA, CDC6, OIP5, and NCAPG) has been established and proposed as a potential prognostic biomarker for LUAD (Li et al., 2018). Although the researchers validated this prognostic model in their hospital, the study did not provide the area under ROC curve AUC values. In our study, the AUC values of the ROC curves were 0.756, 0.753, and 0.705. To screen the filtered genes, we leveraged eight machine learning algorithms and identified three hub genes—HLF, HPCAL1, and NUPR1—which were used to construct a prognostic risk model. Similar to our study, Wu et al. developed a robust four-gene prognostic model that revealed these genes function as tumor suppressors in LUAD, with their high expression predicting a lower risk of mortality (Wu et al., 2022). Notably, their model included HLF, one of the hub genes identified in our study, further validating the reliability of our findings. Furthermore, we analyzed the distribution differences of RiskScore across various clinical parameters and found that RiskScore showed superior prognostic value compared to clinical factors like age and tumor stage. Additionally, univariate Cox regression analysis was carried out to identify prognosis-related factors, leading to the construction of a nomogram model capable of accurately predicting 1-year, 3-year, and 5-year overall survival. Moreover, in terms of the number of genes used, our study employed fewer genes compared to previous reports. This reduction in gene number may facilitate subsequent clinical translation and the development of diagnostic tools, ultimately promoting clinical applications.

HLF belongs to the family of transcription factors known as the proline and acidic amino acid-rich basic leucine zipper (PAR bZIP), which also contains the thyrotroph embryonic factor (TEF) and albumin D-site-binding protein (DBP) (Hunger et al., 1992). Dysregulation of HLF has been observed in various cancer types, with its role varying depending on the biological context, functioning as either an oncogene or a tumor suppressor gene. In ovarian tissue and ovarian stem cells, HLF expression is upregulated, promoting ovarian cancer stem cell properties, proliferation, as well as metastasis (Han et al., 2023). In triple-negative breast cancer (TNBC), HLF enhances proliferation, metastasis, cisplatin resistance, and ferroptosis resistance by activating γ-glutamyl transferase 1 (Li et al., 2022). However, in gliomas and LUAD, HLF functions as a tumor suppressor. Studies have suggested HLF expression is remarkably downregulated in LUAD and is related with a better prognosis (Wang et al., 2021). Furthermore, HLF is related to multiple biological processes, including apoptosis, cell cycle regulation, EMT, and hormone-related pathways such as androgen receptor (AR) and estrogen receptor (ER) signaling. Additionally, HLF is implicated in critical oncogenic pathways, such as the PI3K/AKT, Ras/MAPK, and receptor tyrosine kinase (RTK) signaling pathways (Ahmadi et al., 2024). As a transcription factor, HLF might potentially modulate ferroptosis sensitivity in macrophages by regulating the expression of genes involved in iron metabolism or antioxidant responses.

HPCAL1, which expresses in the plasma membrane and is also known as a visinin-like protein-3 (VILIP-3), is a member of the neuron-specific calcium-binding protein family (Wang L. et al., 2023). Recently, Chen et al. (Wang L. et al., 2023) reported HPCAL1 is a novel driver of autophagy-dependent iron death. It is widely distributed in various human tissues and is expressed in various tumor tissues. The results of existing studies are inconsistent regarding the role of HPCAL1 in tumor growth. The HPCAL1-induced iron death was able to inhibit tumor growth, as found by Chen et al. (2023). HPCAL1 promotes tumor growth in NSCLC and promotes proliferation in glioblastoma, the latter occurring through activation of the Wnt/β-catenin signaling pathway (Zhang D. et al., 2019; Wang et al., 2022). Therefore, the mechanism and function of HPCAL1 in the regulation of tumors require further investigation. The identification of HPCAL1 as a high-risk associated gene in this study suggests a predominantly pro-tumorigenic role in LUAD. Potential mechanisms could involve its participation in the ferroptosis process through the regulation of autophagy or calcium signaling pathways.

NUPR1 is a member of AT hook-containing chromosomal DNA-binding proteins that was first identified and cloned in a study of pancreatitis-induced tissue injury (Mallo et al., 1997). Over the past 2 decades, NUPR1 has been clearly indicated to play a critical role in the development and progression of several cancers, and is also closely associated with a variety of other pathological states such as pancreatitis, diabetes mellitus, neurological disorders, and inflammatory disorders (Martin et al., 2021). NUPR1 prevents the onset of iron death or tissue damage by directly up-regulating the expression of LCN2 (Liu J. et al., 2021). In a cohort study, Tao et al. found NUPR1 acted as a protective factor in the survival prognosis of LUAD (Shu et al., 2022). Furthermore, Zhang et al. revealed that macrophages were the immune cells type most strongly associated with NUPR1 expression in bladder cancer (BLCA) (Zhang et al., 2023). Therefore, it becomes particularly important to study the effect of iron death-related genes in macrophages on tumors. GOKEGG analysis corroborates the involvement of these three core genes in transcriptional regulatory activities, implying that they may exert their functions by altering gene expression profiles.

TME has a crucial role in tumor initiation and progression. Analysis using the ‘CIBERSORT’ and ‘ssGSEA’ algorithms revealed significant differences in immune cell proportions between the HRG and LRG, particularly in macrophages, eosinophils, and mast cells. Moreover, the TMB in the HRG was remarkably higher than that in the LRG (p < 0.001), and GSEA demonstrated significant enrichment of multiple tumor-related pathways in the HRG, such as epithelial-mesenchymal transition (EMT) and cell cycle checkpoint pathways, the activation of which is commonly associated with tumor progression and unfavorable prognosis. Although a direct link between these pathways and the regulation of macrophage ferroptosis by the core genes was not established in this study, they might collectively influence the tumor microenvironment and impact disease progression, thereby highlighting a direction for future investigation. These findings suggest tumors in the HRG are typically associated with enhanced immune evasion mechanisms, enabling them to evade immune surveillance and attack by the host immune system. Studies have shown that tumor-associated macrophages (TAMs) derived from malignant ascites of gastric cancer exhibit a predominant M2 phenotype, which is closely related with poor prognosis in gastric cancer (Eum et al., 2020). Consequently, TAMs in the HRG tend to polarize toward the M2 phenotype, which suppresses T-cell activity by secreting immunosuppressive factors like IL-10 as well as TGF-β, thereby promoting tumor cell proliferation and metastasis (Biswas et al., 2013). The role of eosinophils in cancer immunity has also gained increasing attention. As early as the late 20th century, reports identified the presence of eosinophils in the peripheral blood of cancer patients. Eosinophils not only participate in the antitumor response triggered by immune checkpoint inhibitors (ICIs) but also interact with lymphocytes and T cells (Grisaru-Tal et al., 2022). A clinical study demonstrated that peripheral eosinophil counts significantly increased following ICI therapy (Rafei-Shamsabadi et al., 2022). Additionally, increased eosinophil counts during ipilimumab treatment have been associated with prolonged survival in melanoma patients (Lang et al., 2018). Mast cells also exhibit a dual role in tumor growth by secreting various mediators that either promote or inhibit tumor progression, depending on the tumor type (Ribatti, 2023). Clinical studies have shown increased infiltration of mast cells and CCR2+ cytotoxic T lymphocytes (CTLs), along with their colocalization, are closely related to favorable postoperative prognosis but not to improved survival following chemotherapy (Fan et al., 2023). Moreover, analysis of immune cell density in the tumor and its surrounding stroma has indicated that mast cell infiltration is significantly associated with recurrence-free survival (RFS) in early-stage LUAD (Kammer et al., 2023). Tumors in the HRG interact with immune cells in the TME through immune evasion mechanisms, thereby escaping host immune surveillance and accelerating tumor progression and metastasis. These findings provide novel insights for optimizing future immunotherapeutic strategies, particularly those targeting immune evasion mechanisms.

This study also compared the differences in IC50 values of chemotherapeutic agents between different risk score groups. The results indicated that the high-risk group exhibited higher sensitivity to the chemotherapeutic agent cyclophosphamide and the targeted therapy crizotinib. In contrast, the LRG demonstrated greater sensitivity to chemotherapeutic agents like vinorelbine, paclitaxel, docetaxel, vinblastine, as well as targeted therapies including erlotinib and gefitinib. These findings suggest the risk score can serve as an effective predictor of the sensitivity of LUAD patients to commonly used chemotherapeutic agents. This discrepancy may be closely related to differences in tumor characteristics, immune microenvironment, and molecular mechanisms between the risk groups. The HRG typically features elevated TMB and enhanced immune evasion mechanisms, which may contribute to increased sensitivity to certain drugs. High-TMB tumors are often associated with immune response activation, which could potentiate the efficacy of specific chemotherapeutic agents. Furthermore, an analysis of immune therapy response revealed that two immune checkpoint genes, BTLA and CD47, were remarkably downregulated in the HRG (p < 0.001). BTLA is widely expressed in the immune system, predominantly on T cells, B cells, macrophages, and dendritic cells, with low-level expression on NK cells. Circulating BTLA has been identified as a blood-based predictive biomarker for immune therapy responses in various cancers (Sordo-Bahamonde et al., 2023). CD47 is ubiquitously expressed on the surface of all examined human and mammalian cells. A study by Yoshida et al. demonstrated in early-stage gastric cancer, the CD47 positivity rate was 49.5%, and the 5-year survival rate of the CD47-positive subgroup was remarkably lower than that of the CD47-negative subgroup (Mittrücker et al., 2014). In non-small cell lung cancer (NSCLC) patients, high CD47 expression has been closely related with poor prognosis (Yang et al., 2021). Therefore, we speculate that these two immune checkpoint genes may serve as potential therapeutic targets for early-stage LUAD immunotherapy.

Despite the meaningful findings obtained in this study, several limitations should be acknowledged. Primarily, this research relies heavily on bioinformatic analysis of public datasets, and a key constraint is the lack of direct experimental validation to confirm the observed associations. Specifically, the precise functions of the three key macrophage-associated ferroptosis-related genes (HLF, HPCAL1, NUPR1) in regulating macrophage ferroptosis, polarization, and their interactions with LUAD cells and chemotherapeutic agents remain to be experimentally confirmed. Furthermore, the elucidation of their exact molecular mechanisms, such as specific signaling pathways or protein interactions, requires further investigation. Secondly, while the prognostic model and nomogram developed herein demonstrated good performance on the existing datasets, their practical clinical utility and generalizability necessitate rigorous evaluation in additional, independent external cohorts, particularly through prospective clinical studies. Concurrently, potential biases from public data representativeness and technical artifacts from bioinformatic analyses are constraints to consider. Moreover, translating these fundamental research findings into clinically viable therapeutic targets remains a long-term and challenging endeavor.

Therefore, future research is crucial to address these limitations and deepen our understanding. Subsequent efforts should focus on functional validation through experimental approaches: (1) Utilizing gene editing (e.g., CRISPR/Cas9), overexpression/knockdown techniques, combined with co-culture systems, to conduct in-depth *in vitro* investigations into the specific mechanisms by which HLF, HPCAL1, and NUPR1 regulate macrophage ferroptosis, polarization, and interactions with LUAD cells; (2) Developing relevant gene knockout or conditional knockout mouse models to validate their actual roles in LUAD development and TME remodeling *in vivo*; (3) Further exploring the upstream and downstream regulatory networks of these core genes to identify potential pharmacological intervention targets. Such follow-up studies will provide more robust support for our findings and facilitate the development of novel LUAD therapeutic strategies based on macrophage ferroptosis.

## 5 Conclusion

This study established a novel prognostic risk model for early-stage LUAD based on three macrophage-related ferroptosis genes. This model effectively evaluates the survival outcomes of early-stage LUAD patients and provides potential biomarkers and therapeutic targets. These findings offer critical theoretical support for targeted therapeutic strategies in early-stage LUAD, particularly those focusing on macrophage ferroptosis.

## Data Availability

The original contributions presented in the study are included in the article/supplementary material, further inquiries can be directed to the corresponding authors.
